# Contribution of Cross-Linker and Silica Morphology on Cr(VI) Sorption Performances of Organic Anion Exchangers Embedded into Silica Pores

**DOI:** 10.3390/molecules25051249

**Published:** 2020-03-10

**Authors:** Ecaterina Stela Dragan, Doina Humelnicu

**Affiliations:** 1 “Petru Poni” Institute of Macromolecular Chemistry, Grigore Ghica Voda Alley 41 A, 700487 Iasi, Romania; 2 Faculty of Chemistry, “Al. I. Cuza” University of Iasi, Bd. 11 Carol I, 700506 Iasi, Romania; doinah@uaic.ro

**Keywords:** anion exchanger, cross-linker, chromium (VI), porous silica, sorption kinetics, sorption isotherm, reusability

## Abstract

Removal of Cr(VI) from the environment represents a stringent issue because of its tremendous effects on living organisms. In this context, design of sorbents with high sorption capacity for Cr(VI) is getting a strong need. For this purpose, poly(vinylbenzyl chloride), impregnated into porous silica (PSi), was cross-linked with either *N*,*N*,*N*’,*N*’-tetramethyl-1,2-ethylenediamine (TEMED) or *N*,*N*,*N*’,*N*’-tetramethyl-1,3-propanediamine, followed by the reaction of the free -CH_2_Cl groups with *N*,*N*-diethyl-2-hydroxyethylamine to generate strong base anion exchangers (ANEX) inside the pores. The PSi/ANEX composite sorbents were deeply characterized by FTIR spectroscopy, SEM-energy dispersive X-ray spectroscopy (EDX), thermogravimetric analysis (TGA), and water uptake. The sorption performances of composites against Cr(VI) were investigated as a function of pH, contact time, initial concentration of Cr(VI), and temperature. It was found that the cross-linker structure and the silica morphology are the key factors controlling the sorption capacity. The adsorption process was spontaneous and endothermic and well described by pseudo-second-order kinetic and Sips isotherm models. The maximum sorption capacity of 311.2 mg Cr(VI)/g sorbent was found for the composite prepared with mesoporous silica using TEMED as cross-linker. The PSi/ANEX composite sorbents represent an excellent alternative for the removal of Cr(VI) oxyanions, being endowed with fast kinetics, equilibrium in about 60 min, and a high level of reusability in successive sorption/desorption cycles.

## 1. Introduction

Nowadays, finding highly efficient systems for the removal of heavy metal ions (HMIs) from the aquatic environment is a stringent issue in front of the scientists all over the world, because they are highly toxic (even at low concentrations), not biodegradable, and have an accumulation tendency in living organisms—most of them being carcinogenic. Synthetic polymeric sorbents [1,2,3,4,5,6] and various biosorbents [7,8,9,10,11,12] are investigated for their performances in the recovery/removal of HMIs. Among the HMIs present in the wastewaters, chromium, especially Cr(VI), is one of the most dangerous contaminants of the surface and ground water, being considered a powerful carcinogenic and teratogenic agent threatening living organisms [1,2,11,13,14,15,16,17,18,19,20,21,22,23,24,25,26,27,28,29]. On the other hand, Chromium(III) is listed as an essential element, as a micronutrient, being involved to maintain the normal metabolism of glucose, cholesterol, and fat in human bodies [1,2], being poisonous only at a high concentration. The main source of chromium into the natural waters are the industrial wastewaters coming from the chrome plating, paints, and pigments production, leather tanning, metal finishing, electroplating industry, and wood preservation. Cr(VI) is highly mobile in the environment, can easily penetrate the cell membrane having a corrosive effect on tissues, including lung cancer, and kidney, liver, and stomach [17]. According to the World Health Organization (WHO) drinking water guidelines, the maximum allowable limit for total chromium is 50 μg/L [1,2]. Therefore, to avoid the dangerous impact of Cr(VI) on human health and on the environment, as well as for economic considerations, it is essential to remove/recover Cr(VI) from the wastewaters before disposal [1,2,3]. Various techniques are available for the removal of Cr(VI) such as ion exchange, chemical precipitation, reduction [13,14], reverse osmosis, foam flotation, electrolysis, membrane filtration [15,16], ultrafiltration [17], sorption, and biosorption [18,19,20,21,22,23,24,25,26,27,28,29,30,31]. Some of these techniques have disadvantages of either producing toxic sludge as is the case of chemical precipitation, or asking for high capital costs (reverse osmosis), recovery of value metal being difficult [27,28]. Therefore, the research interest is lately concentrated more on the sorption methods because they offer high efficiency and selectivity, are easy to handle, the metal can be recovered, the sorption process is reversible, and it is cost-effective. In aqueous environment, at pH > 2.0, Cr(VI) exists as oxyanion, and that is the reason why ligands carrying a positive charge, generated by protonation or quaternization, have been successfully used—sorption by electrostatic attractions being favored [21,22,23,24,25,26,27,28,29,30]. Synthetic sorbents offer the possibility to introduce almost any reactive functionality, which could be involved in the retention of HMIs. Thus, Zhang et al. have developed a composite sorbent consisting of a weak base anion exchanger having tertiary amine groups (D301) modified by poly(epichlorohydrin-dimethylamine) with a maximum sorption capacity of 194 mg Cr(VI)/g [22]. Macroporous strong base anion exchangers (SBAE) bearing quaternary ammonium salt groups have been synthesized and successfully used by our group in the removal of Cr(VI) either as single anion exchangers [25] or embedded in a composite consisting of chitosan and poly(vinyl amine) as cryobeads [24], their maximum sorption capacity being of 200–320 mg Cr(VI)/g sorbent. A biosorbent consisting of chitosan beads modified with malic acid by Zhang et al. have showed a maximum equilibrium sorption capacity of 383.2 mg Cr(VI)/g [29].

It was reported that organic ion exchangers have usually high ion exchange capacities, but low mechanical strength, slow kinetics, and present multiple variation of volume during the ion exchange processes. Therefore, the scientific and technological interest is lately focused on designing novel sorbents endowed with fast adsorption/desorption rate of Cr(VI) oxyanions, high mechanical strength, selectivity, and high reusability. These requirements could be successfully fulfilled by organic/inorganic composites [18,19,31,32,33,34,35,36,37,38,39,40,41]. Thus, magnetic composite sorbents, which allow their easy magnetic separation from the aqueous environment, have been recently reported [31,33,34]. Graphene oxide (GO) and reduced GO represent other attractive inorganic constituents of the composite sorbents used in the removal of Cr(VI) [19,35,38].

Silica/organic polymer composites have been recently promoted for the removal of Cr(VI) due to their performances proved by the mechanical resistance in the successive sorption/desorption cycles of heavy metal ions [39,41]. Composite ion exchangers, having silica as a core coated with organic polymer layers, are endowed with a fast uptake of the ionic solutes and mass transfer properties more favorable for high-performance chromatographic separations [42,43], but their sorption capacity for ionic species is low in many cases, because most of the composite is occupied by the inorganic component. To remediate this limitation, various organic ionic polymers have been lately generated inside the silica pores [44,45,46]. The exchange kinetics and the dimensional stability of the ion exchangers during the multiple sorption/desorption cycles have been thus improved [44,45].

In our previous work we have synthesized novel composite sorbents by cross-linking poly(vinylbenzyl chloride) (PVBC), impregnated into silica pores, with *N*,*N*,*N*’,*N*’-tetramethyl-1,3-propanediamine (TMPDA), followed by the reaction of the free -CH_2_Cl groups with *N*,*N*-diethyl 2-hydroxyethylamine (DEHEA) [47]. These composite sorbents demonstrated excellent sorption capacity and selectivity for methyl orange, as well as high reusability. Our hypothesis for the present study is that such composite sorbents endowed with a high stability of the particle size and a high number of quaternary ammonium groups could represent an alternative for the sorption Cr(VI) as oxyanions. The adsorption behavior of such composite sorbents toward Cr(VI) has never been accomplished until now. Furthermore, it was assumed that the structure of the tertiary diamine used as cross-linker could influence the sorption performances of the composite sorbents. Therefore, another cross-linker was used in this work, i.e., *N*,*N*,*N*’,*N*’-tetramethyl-1,2-ethylenediamine (TEMED). The composite sorbents investigated in this work were abbreviated as PSi.1/ANEX1, PSi.1/ANEX2, and PSi.2/ANEX2, which means: PSi-porous silica, 1 or 2; ANEX-for anion exchanger, followed by 1, when the cross-linker used was TEMED, or 2, when the cross-linker was TMPDA. The adsorption performances of the composite sorbents toward Cr(VI) were systematically investigated as a function of pH, contact time, initial concentration of Cr(VI), temperature.

## 2. Results and Discussion

### 2.1. Synthesis of PSi/ANEX Composite Sorbents

**Scheme 1:**
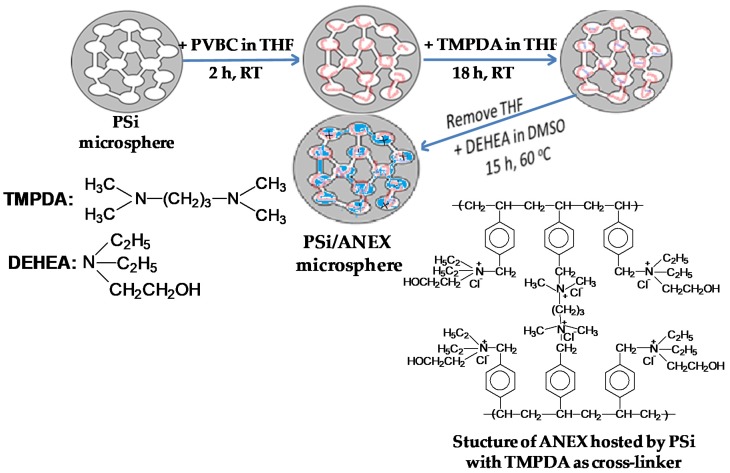
Synthesis of porous silica (PSi)/anion exchangers (ANEX) composite by impregnation first of poly(vinylbenzyl chloride) (PVBC) into PSi pores followed by cross-linking with TEMED, and then generation of the quaternary ammonium salt groups by the reaction of the free -CH_2_Cl groups with *N*,*N*-diethyl 2-hydroxyethyl amine (DEHEA); RT-room temperature.

Three PSi/ANEX composite sorbents were synthesized in this work (PSi1/ANEX1, PSi1/ANEX2, and PSi2/ANEX2) by the generation of SBAEs into the pores of two kinds of porous silica: a mesoporous silica, coded with PSi1, and a macroporous silica, coded with PSi2. From the N2 adsorption isotherms (Figure S1a,b), the following values were found for their textural characteristics: S_sp_ of 95.097 m^2^/g and pore volume of 0.332 cm^3^/g (for pores with diameter < 45.5 nm), for PSi1; S_sp_ of 23.963 m^2^/g and pore volume of 0.0337 cm^3^/g (for pores with diameter < 46.9 nm), for PSi2. As cross-likers for PVBC, two ditertiary amines were used: TEMED and TMPDA, at a mole ratio of 0.2 moles diamine: 1 mole -CH_2_Cl. The -CH_2_Cl groups remained after cross-linking were used for the generation of (vinylbenzyl *N*,*N*-diethyl 2-hydroxyethyl) ammonium chloride units by the reaction with DEHEA in excess. Scheme 1 presents the strategy for the synthesis of the PSi/ANEX composites.

The PSi/ANEX composites thus prepared are very rich in quaternary ammonium salt groups, which are expected to electrostatically bind Cr(VI) oxyanions. The PSi/ANEX composites were characterized by FTIR spectroscopy, TGA and SEM-EDX.

### 2.2. Characterization of PSi/ANEX Composites

#### 2.2.1. Structural Characterization

**Figure 1:**
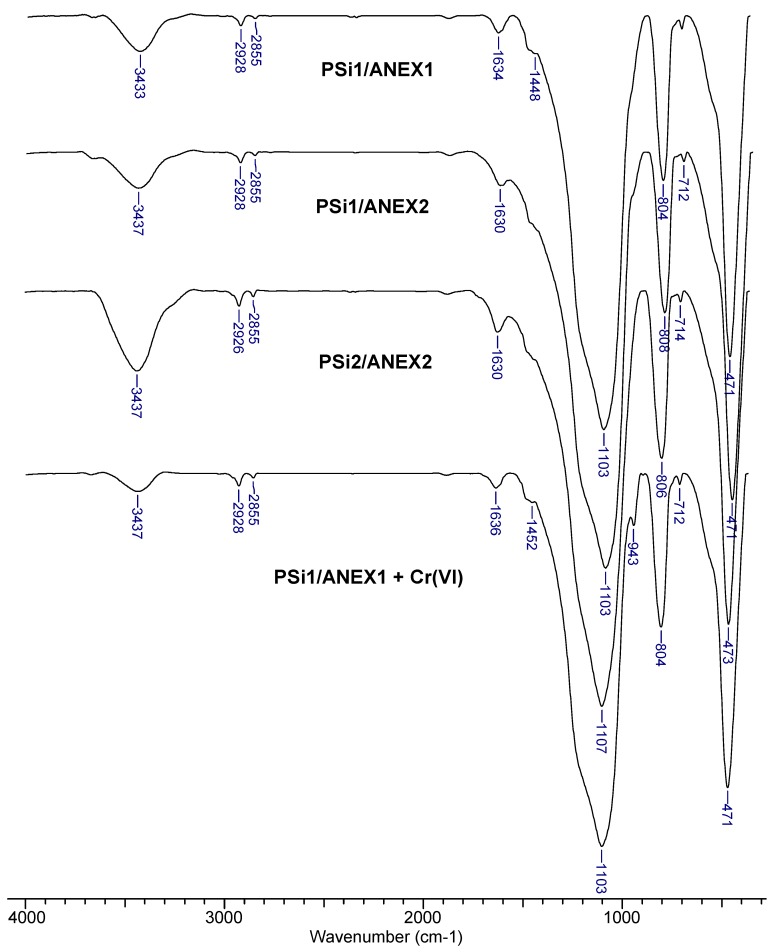
FTIR spectra of the PSi/ANEX composites before loading with Cr(VI) and of the composite PSi1/ANEX1 after loading with Cr(VI).

Structural characterization of the bare PSi and of the PSi/ANEX composites was performed by FTIR spectroscopy, the spectra being presented in Figure S2 and Figure 1, respectively.

As can be seen in Figure 1, the following peaks are visible in all FTIR spectra of the composites: around 3437 cm^−1^, assigned to the stretching vibrations of -OH groups from silica and from ANEX; 2927 and 2854 cm^−1^ attributed to the -CH_3_ and -CH_2_-CH_3_ groups from ANEX; the shoulder at around 1450 cm^−1^ attributed to the stretching vibrations of -CH_2_- groups; the peaks at 1633 cm^−1^ in PSi1/ANEX1, and at 1629 cm^−1^ in the spectra of PSi1/ANEX2 and PSi2/ANEX2, are assigned to in-plane bending of the primary OH from the substituents -CH_2_-CH_2_-OH in quaternary ammonium salt groups. The peak at around 712 cm^−1^, visible in all spectra, confirms the presence of ANEX, attributed to the stretching vibrations of aromatic ring (in PVBC). The peaks located at around 1103, 804 and 470 cm^−1^ in all spectra were assigned to the asymmetric stretching vibration of Si–O–Si bonds, bending vibrations of Si–O bonds, and deformation out-of-plane vibrations of Si–O bonds, respectively. In the FTIR spectra of PSi1 and PSi2, presented in Figure S2, these peaks are located at 1088, 817, and 470 cm^−1^. Even if the silica main peaks are screening the peaks of the organic ANEX, after loading with Cr(VI) (Figure 1), a new peak is visible at 943 cm^−1^, which could be assigned to νCr–O bonds [24,48].

#### 2.2.2. Thermogravimetric Analysis (TGA)

Thermal behavior of the composites allow to establish the real content of organic part in each composite and to discriminate them from the thermal feature. Therefore, the content in ANEX of all composites was evaluated by TGA. Figure S3 shows that no weight was lost from the pristine PSi2, taken as an example, up to 700 °C.

**Figure 2:**
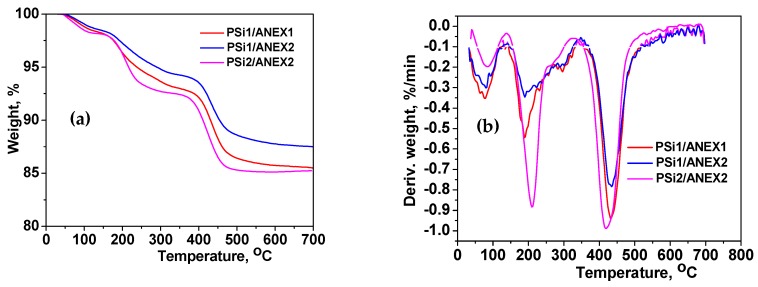
TG (**a**) and DTG (**b**) curves of the PSi/ANEX composite sorbents.

Figure 2 presents the TG (2a) and DTG (2b) curves characteristic for the thermal degradation of the three samples of PSi/ANEX. As Figure 2 shows, all PSi/ANEX composites present four stages for their thermal degradation. The first stage, with a weight loss of 1.64%, 1.57%, and 1.92%, for PSi1/ANEX1, PSi1/ANEX2, and PSi2/ANEX2, respectively, corresponds to the loss of residual water. The second stage, with a maximum located at 190.02 °C, 190.15 °C, and 207.96 °C, and a weight loss of 4%, 2.55%, and 4.39%, found for PSi1/ANEX1, PSi1/ANEX2, and PSi2/ANEX2, respectively, was attributed to the dealkylation of the quaternary ammonium salt groups; the third stage with a maximum located at 296.14 °C, 290 °C, and 266 °C, and a weight loss of 1.39%, 1.6%, and 1.41%, found for PSi1/ANEX1, PSi1/ANEX2, and PSi2/ANEX2, respectively, was ascribed to the degradation of the cross-linking between the main chains; the main stage of thermal degradation is the fourth stage, with the peaks located at 432.34 °C, 435.45 °C, and 420 °C, the weight loss being of 7.45%, 6.85, and 7.12%, found for PSi1/ANEX1, PSi1/ANEX2, and PSi2/ANEX2, respectively, this stage being assigned to the degradation of the main chains. Taking into account the weight loss in the second, the third and the fourth stages, the amount of ANEX in each composite would be of 12.84%, 11%, and 12.92%, in PSi1/ANEX1, PSi1/ANEX2, and PSi2/ANEX2, respectively.

#### 2.2.3. Morphological and Textural Characterization

**Figure 3:**
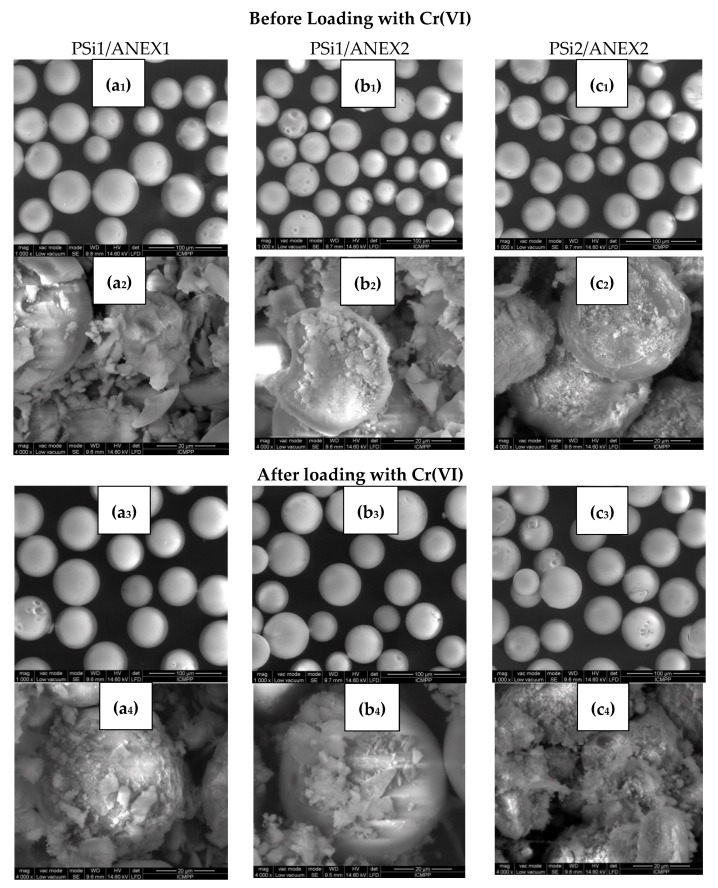
SEM images of the exterior (mag 1000×), and the interior morphology (mag. 4000×) of the PSi/ANEX composites before loading with Cr(VI) of the PSi1/ANEX1 (**a_1_** and **a_2_**), PSi1/ANEX2 (**b_1_** and **b_2_**), PSi2/ANEX2 (**c_1_** and **c_2_**) composites, and after loading with Cr(VI) of PSi1/ANEX1 (**a_3_** and **a_4_**), PSi1/ANEX2 (**b_3_** and **b_4_**), and PSi2/ANEX2 (**c_3_** and **c_4_**).

By SEM analysis, information about the external and internal morphology of the PSi/ANEX composites were obtained. The SEM images before and after loading with Cr(VI) for the three composites are presented in Figure 3. As Figure 3a_1_–c_1_ show, there are not significant differences between the three composite microspheres as from the external point of view. However, the silica porosity has a strong influence on the internal morphology visible into the broken PSi/ANEX composites (see Figure 3a_2_–c_2_).

As can be seen, the organic part is difficult to be identified in the two composites based on mesoporous silica (PSi1/ANEX1 and PSi1/ANEX2), while the larger pores of macroporous silica (PSi2/ANEX2) give the possibility to identify the organic material entrapped into the silica pores (Figure 3c_2_). The SEM images of the internal morphology of the composites after their loading with Cr(VI), presented in Figure 3a_4_–c_4_ support the strong interaction between the ANEX and metal oxyanions by the more compact surfaces. The EDX profiles of the three PSi/ANEX composites are presented in Figure S3. As can be seen the content in nitrogen is in the range 2.95–3.85, and that of chlorine in the range 0.77–1.32.

As Figure S1c,d shows, the textural characteristics of porous silica were also changed after the synthesis of ANEX inside the pores. Thus, the values of S_sp_ decreased from 95.097 m^2^/g, found for PSi1, to 84.463 m^2^/g found for PSi1/ANEX2, and from 23.963 m^2^/g, found for PSi2, to 14.831 m^2^/g found for PSi2/ANEX2. These changes support the presence of the ANEX inside the silica pores.

The values of water uptake of (WU) of pristine PSi and of the PSi/ANEX composites are presented in Table S1. As ca be seen, the WU decreased after the synthesis of ANEX inside the silica channels, more in the case of PSi1/ANEX1 (0.9271 g/g) than in the case of the other two composites (1.281 g/g and 1.176 g/g for PSi1/ANEX2 and PSi2/ANEX2, respectively). This lower value of WU in the case of the former sorbent could be attributed to a higher density of hydrophobic substituents in this case, which could also explain the higher sorption capacity for Cr(VI) oxyanions.

#### 2.2.4. Determination of pH_PZC_

**Figure 4:**
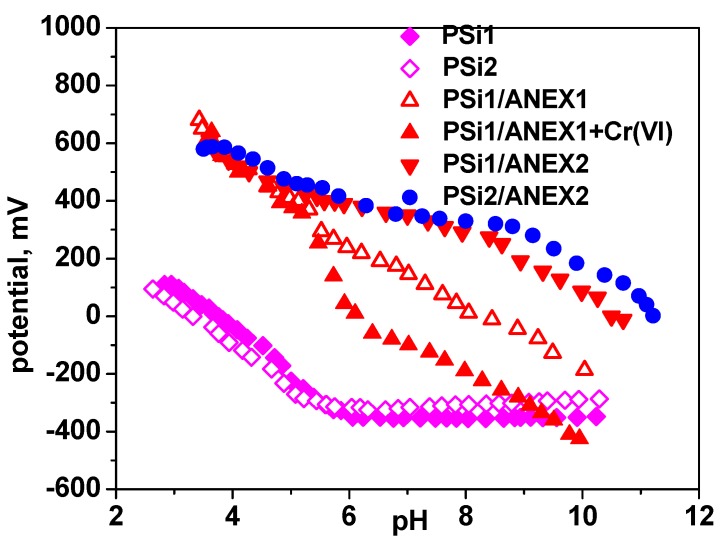
Potentiometric titrations of bare silica microspheres (PSi1 and PSi2) and of the PSi/ANEX composites. pH_PZC_ for PSi1/ANEX1 has been measured both before (empty triangle) and after loading with Cr(VI) (filled triangle).

As Figure 4 shows, the pH_PZC_ values of both PSi1 and PSi2 are situated at pH 3.32 and 3.7, respectively. By the construction of the strong base anion exchangers inside the silica pores, the values of pH_PZC_ dramatically increased up to 8.2, 10.5, and 11.2 for PSi1/ANEX1, PSi1/ANEX2 and PSi2/ANEX2, respectively.

Potentiometric titration curves of PSi1/ANEX1, before and after loading with Cr(VI) shows that pH_PZC_ decreased from 8.2 to about 6 after the sorption of Cr(VI) oxyanions. This demonstrates the strong interaction between the composite sorbent having quaternary ammonium salt groups and the sorbate anions.

### 2.3. Sorption of Cr(VI) onto PSi/ANEX Composites

#### 2.3.1. pH Effect

**Figure 5:**
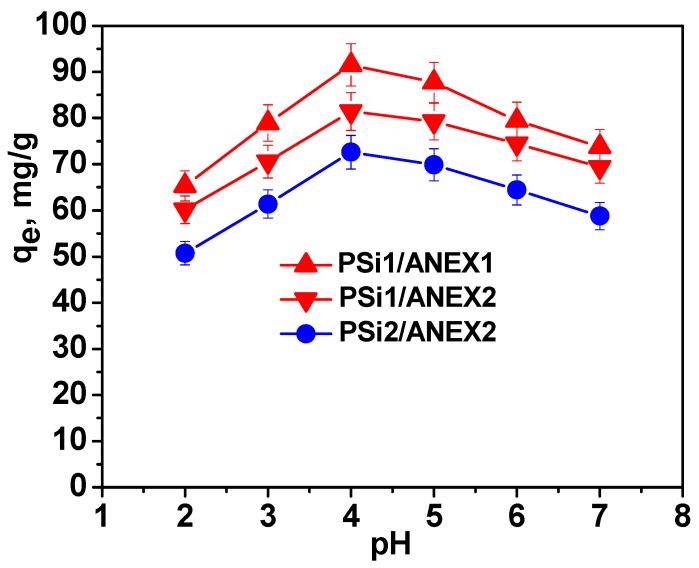
Effect of pH on the sorption capacity (q_e_) of the SiO_2_/ANEX composite sorbents for Cr(VI) oxyanions: sorbent dose 1 g/L; temp. 23 °C; contact time 6 h; shaking rate 180 r.p.m.

Initial solution pH is one essential parameter that strongly influences the performances and the sorption mechanism of a sorbent because it determines the sorbent surface charge and the metal ion species in solution [7,10,26,32,38,49]. The effect of solution pH on the Cr(VI) removal by the PSi/ANEX composites is shown in Figure 5.

As can be seen in Figure 5, all composite sorbents display the same trend when the pH increased in the range 2–7. Thus, the q_e_ values abruptly increased when pH increased from 2 to 4 and gradually decreased with the increase of pH up to 7, as follows: for PSi1/ANEX1, the sorption capacity increased from 65.29 mg/g at pH 2, up to 91.54 mg/g at pH 4; from 60.15 mg/g up to 81.43 mg/g, for PSi1/ANEX2, and from 50.74 mg/g up to 72.62 mg/g, in the case of PSi2/ANEX2, for the same pH values. However, the decrease of q_e_ was not so dramatic when the pH increased from 4 to 7, the values being still 73.81 mg/g, 69.38, and 58.76, for PSi1/ANEX1, PSi1/ANEX2, and PSi2/ANEX2, respectively. The effect of pH on the removal of Cr(VI) by the PSi/ANEX composites is almost similar with that reported for other sorbents containing quaternary ammonium salt groups, which could electrostatically interact with Cr(VI) oxyanions [21,24,27,28]. The explanation for this behavior is based on the ionic species present in solution as a function of pH. When the H^+^ are in excess, Cr(VI) can exist as several oxyanions (Cr_2_O_7_^2^^−^, HCr_2_O_7_^−^, HCrO_4_^−^, and CrO_4_^2^^−^) [19,22,37,38,49]. The majority of the sorbents tested for the removal of Cr(VI) have an optimum pH located in the range 2–3. However, at pH 1.0, the uptake capacity is low because the chromium is predominantly present as H_2_CrO_4_, a strong competition between H_2_CrO_4_ and protons for adsorption sites being possible [50]. From literature, it is known that the main possible interactions between an ion exchanger and the ions in solutions are ion exchange and chelation, depending on the type of functional groups. When the main functional groups involved in the sorption process are amines [19,22,32,37,48,49,50], or nonionic polymers [41], after a maximum of sorption located at pH about 2, a dramatic decrease of the removal efficiency of chromium has been reported. Increasing the pH, the OH^−^ ions are present at higher concentration, and compete with metal oxyanions for the sorption sites of the composite sorbent, the sorption capacity and removal efficiency of chromium decreasing accordingly [34,50]. Concerning the sorption of Cr(VI) onto the composite sorbents under study in this work, at pH located in the range 2–6, Cr(VI), which can exists as HCr_2_O_7_^−^ or HCrO_4_^−^, will need one site on the composite sorbent as the adsorption process to occur, the reaction being described by the following Equation (1) [21,22]:

RCl + HCrO_4_^−^ = R^+^ HCrO_4_^−^ + Cl^−^ (1)

At pH > 6, the most part of Cr(VI) exists as Cr_2_O_7_^2−^ and CrO_4_^2−^, and these divalent oxyanions need two sites on the sorbent to realize the sorption, the reaction taking place according to the following Equation (2) [22]:

2RCl + CrO_4_^2−^ = R_2_^2+^ CrO_4_^2−^ + 2Cl^−^ (2)

This explains why the sorption capacity decreases at pH > 6. Increasing further the pH, the OH^−^ ions competed with Cr(VI) oxyanions, and the sorption capacity dramatically decreased. This sorption mechanism has been reported for other sorbents containing quaternary ammonium salt groups [21,24,27,28].

#### 2.3.2. Kinetic Study

**Figure 6:**
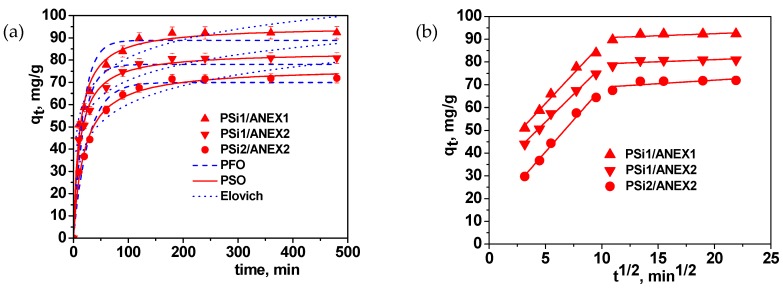
(**a**) Sorption kinetics of Cr(VI) onto PSi/ANEX composite microspheres fitted with three kinetic models: (**b**) Fitting IPD model on the sorption kinetics data of Cr(VI) onto the PSi/ANEX composites: sorbent dose 1 g/L; temp. 23 °C; initial pH 4.0; initial concentration of Cr(VI) 100 mg/L.

The effect of contact time on the sorption of Cr(VI) oxyanions by the PSi/ANEX composites is presented in Figure 6.

Figure 6a presents the experimental values of q_t_ as a function of time, fitted with three kinetic models, pseudo-first order (PFO), pseudo-second order (PSO), and Elovich kinetic models, the equations corresponding to each model being presented in Table S2. The kinetic parameters obtained by fitting four kinetic models on the experimental data are presented in Table 1.

As can be seen in Table 1, the kinetic data for the sorption of Cr(VI) onto the composite sorbents were more accurately described by the PSO and Elovich kinetic models, the values of R^2^ and of the χ^2^ being higher and respectively lower for these models than for the PFO kinetic model. The calculated values of q_e_ (q_e_ calc.), were also very close to the experimental q_e_ (q_e_, exp). These results would indicate the chemisorption as possible mechanism of sorption, but as it was already shown in Section 2.3.1. (the effect of pH, Figure 5), the most probable mechanism of sorption is the ion exchange between the charged sites of the strong base anion exchanger hosted in the porous silica and the Cr(VI) anionic species present in solution, as a function of pH. In the case of chemisorption, the sorption process is dependent on the sharing of electrons between the adsorbate and the surface of the prepared adsorbents and the availability of sorption sites [25,32,47,51,52,53], but it is not the case of the composite sorbents described in this work (Scheme 1). The well-fitting of the experimental data by the Elovich model indicates the heterogeneity of adsorbent surfaces [32,47]. The values of Elovich parameter α for PSi1/ANEX1 is higher than that of PSi1/ANEX2 and much higher than that of PSi2/ANEX2, demonstrating that, the number of sites available for adsorption in the first composite sorbent is higher than that in the PSi1/ANEX2, and much higher than in PSi2/ANEX2.

In an agitated system, the transport of the metal ions through the boundary layer to the sorbent surface, and the transport of solute from the sorbent surface into the pores, called intra-particle diffusion, or inner diffusion, are the most important steps. The intra-particle diffusion (IPD) model proposed by Weber and Morris is used to explain the diffusion mechanism of the adsorption process (see equation which describes the IPD model, Table S2). C_i_ term in the Weber and Morris equation gives information about the thickness of boundary layer, which means the larger the intercept, the greater the contribution of the film diffusion in the rate limiting step [32,47,49,54]. If the plot of q_t_ vs t^0.5^ gives a straight line, which passes through the origin, then the sorption process is controlled only by IPD process. As can be seen in Figure 6b, the plots of q_t_ vs t^0.5^ for the PSi/ANEX composite sorbents are two-linear: the first region could be ascribed to the external resistance to mass transfer. The second linear part is attributed to the IPD or pore diffusion. In this region, the decrease in the concentration of metal ions, as well as the diminution of the sites available for adsorption took place. Hence, even if the adsorption process involved IPD, this is not the only rate-controlling step. The values of k_id_, C_i_, and R^2^ corresponding to the first region in Figure 6b are listed in Table 1. As can be seen, the highest value of C_i_ was found for the PSi1/ANEX1 (35.42 mg/g), the lowest for PSi2/ANEX2 (12.36 mg/g), the value for PSi1/ANEX2 being in the middle (29.205 mg/g). These values indicate that the role of film diffusion is more important in the case of PSi1/ANEX1 than in the case of PSi1/ANEX2, and much larger than for PSi2/ANEX2. The morphology of the pristine silica (PSi1—mesoporous, and PSi2—macroporous) could have also a contribution. The high values of k_id.1_ obtained by using PSi/ANEX composites indicate that these composite sorbents have a fast removal rate for Cr(VI) from aqueous solution.

#### 2.3.3. Adsorption Isotherm Models

**Figure 7:**
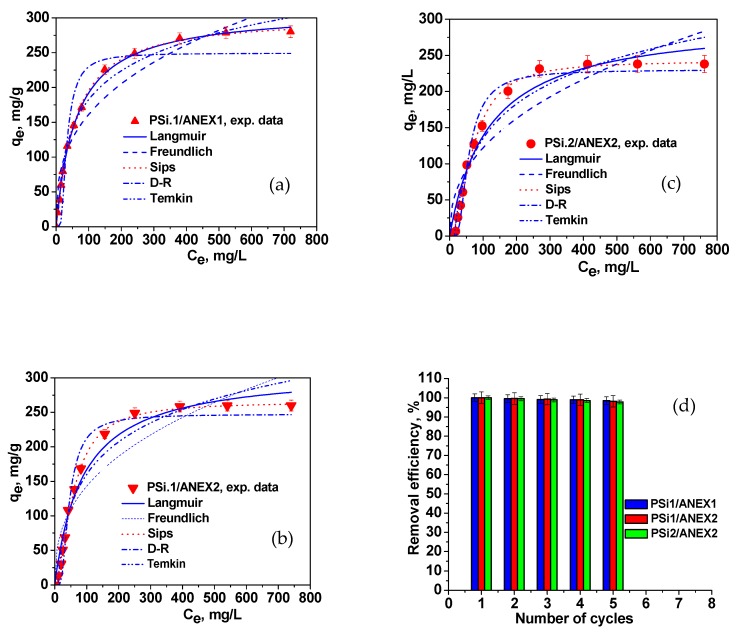
Sorption isotherms of Cr(VI) onto PSi.1/ANEX1 (**a**), PSi.1/ANEX2 (**b**), PSi.2/ANEX2 (**c**), and removal efficiency as a function of number of sorption/desorption cycles (**d**); sorption conditions: sorbent dose 1 g/L, pH 4, temperature 23 °C, contact time 6 h; desorption conditions: 0.1 M NaOH, 6 h, pH 4.

The experiments of sorption at equilibrium were conducted at 23 °C with all PSi/ANEX composites, the experimental data for the adsorption capacity at equilibrium (q_e_) as a function of the Cr(VI) concentration in solution at equilibrium and their modeling with five isotherm models are presented in Figure 7a–c. The non-linear forms of the equations corresponding to the isotherm models can be seen in Table S3.

The isotherm parameters obtained by modeling the experimental data of sorption at equilibrium are presented in Table 2. As can be seen in Figure 7a–c, the sorption process of Cr(VI) oxyanions is described by L type isotherms, on all composite sorbents. As Table 2 shows, the most suitable isotherm models, which describes the sorption of Cr(VI) are Sips and Langmuir, the values of R^2^ and χ^2^ being the highest and, respectively, the lowest for Sips isotherm than for Langmuir model. The values of the maximum sorption capacity given by Sips isotherm allow to discriminate the influence of the cross-linker structure and of silica morphology, being the highest for PSi1/ANEX1 and the lowest for PSi2/ANEX2. This shows that TEMED led to an ANEX with a higher sorption capacity than DMPDA and the mesoporous silica (PSi1), having a higher surface area, was more efficient than the macroporous silica (PSi2) in the sorption of Cr(VI) oxyanions.

Based on the values of K_L_ (Table 2) and the initial concentration of metal ions, the values of constant separation factor (R_L_, Table S3) were calculated for the initial concentrations varying in the range 25–1000 mg/L. As can be seen in Table 2, the values of R_L_ for all sorbents lies in the range 0 < R_L_ < 1, supporting the suitability of the PSi/ANEX composite sorbents for the removal of Cr(VI) from the aqueous solutions. The values of 1/*n* < 1 in the Freundlich isotherm show the bond energies increase with the surface density and support the feasibility of sorption for all composites.

Modeling the adsorption data with D-R isotherm allows the calculation of the mean free energy of adsorption, E, which values give information about the sorption mechanism [8,24,32,47]. The values of E, which magnitude is higher than 40 kJ·mol^−1^ for PSi1/ANEX1 and PSi1/ANEX2, and about 36 kJ·mol^−1^ for PSi2/ANEX2, would indicate chemisorption as possible mechanism of Cr(VI) sorption. However, for these composite sorbents, the ion exchange seems to be the most probable sorption mechanism (Section 2.3.1 and Section 2.3.2).

The Temkin constant, b_T_, related to heat of sorption of Cr(VI) oxyanions were 40.52 J/mol, 35.69 J/mol, and 35.15 J/mol for PSi1/ANEX1, PSi1/ANEX2, and PSi1/ANEX2, respectively.

The maximum sorption capacity for Cr(VI) of the PSi1/ANEX1 composite given by the Langmuir isotherm was compared in Table 3 with the values recently reported in literature for other sorbents.

As can be observed, by the value of q_m_, the novel composite sorbent designed in this work is situated among the best composite sorbents, making it a promising sorbent for the removal of Cr(VI) from aqueous solution.

#### 2.3.4. Reusability

The regeneration in convenient conditions and the level of reusability of the composite sorbents are two characteristics of paramount significance when the feasibility of a sorbent is under study and therefore they must be investigated. The behavior of all three sorbents during five consecutive sorption/desorption cycles of Cr(VI) can be seen in Figure 7d. The fast desorption of the metal ions in one step with 0.1 M NaOH and the almost integral preservation of the sorption capacity at the end of the fifth cycle recommend these composites as promising sorbents for the removal of Cr(VI) as oxyanions.

#### 2.3.5. Thermodynamics

To get further information on the sorption mechanism of Cr(VI) oxyanions onto the PSi/ANEX composites, the thermodynamic parameters (ΔG°, ΔH°, and ΔS°) have been evaluated from the values of the sorption equilibrium constant (*K_c_*) calculated at five temperatures in the range 298 K–318 K, with Equation (3).

(3) $$K_{c}=\frac{q_{e}}{C_{e}}$$

where: q_e_ is the amount of Cr(VI) sorbed at equilibrium, mg/g, and C_e_ is the concentration of metal ions at equilibrium in aqueous phase, mg/L.

According to Equation (3), the units of K_c_ are L/g, but it should be dimensionless [51,56,57]. To solve this problem, the concentration of Cr(VI) was transformed in molar form, and, according to Tran et al., considered the standard state C^o^ = 1 mol/L [51,56,57].

**Figure 8:**
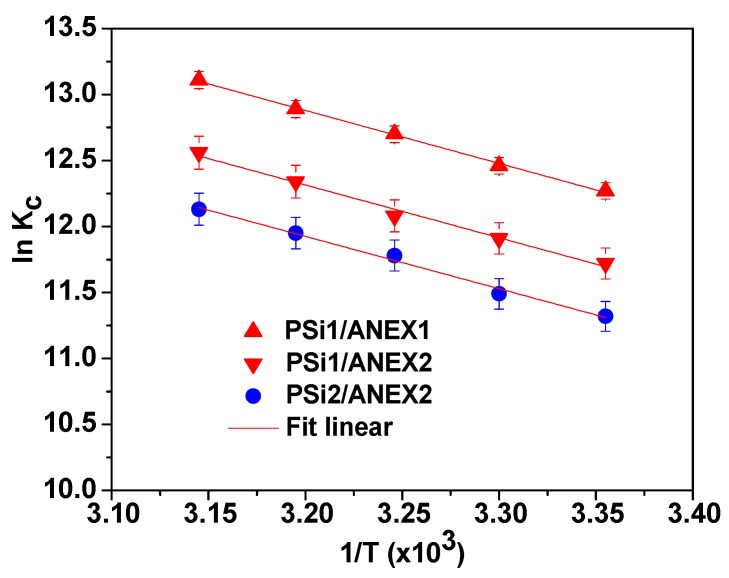
Plot of ln K_c_ versus 1/T for the sorption of Cr(VI) ions onto the porous silica/ANEX composite sorbents; sorption conditions: sorbent dose 1 g/L, pH 4, C_in_ = 100 mg/L, contact time 6 h.

The values of enthalpy (ΔH°) and entropy change (ΔS°) for the sorption of Cr(VI) ions onto the PSi/ANEX composite sorbents were evaluated using Van’t Hoff equation (Equation (4)) from the slope and intercept of the plot lnK_c_ vs. 1/T (Figure 8):

(4) $$\ln K_{c}=\frac{\Delta S^{{}^{\circ}}}{R}-\;\frac{\Delta H^{{}^{\circ}}}{\mathrm{RT}}$$

The change in the standard Gibbs free energy ΔG° was calculated with Equation (5):

(5) $$\Delta G^{{}^{\circ}}=\;-\mathrm{RT}\ln K_{C}$$

The thermodynamic parameters for the sorption of Cr(VI) ions onto the three composite sorbents are presented in Table 4.

The negative values of ΔG° show that the sorption process was spontaneous, and the increase of the negative value with the increase of temperature supports the increase of the degree of spontaneity for the sorption of Cr(VI) ions onto all PSi/ANEX composites. The most negative values of ΔG° found for Psi.1/ANEX1 support the highest amount of metal ions sorbed at equilibrium (Table 2). The positive values of ΔH° show the sorption process was endothermic, and the positive values of ΔS° indicate the increase of the randomness at the solid−liquid interface [19,21,24,36].

## 3. Materials and Methods

### 3.1. Materials

Two sorts of porous silica, different by their morphological characteristics were purchased from the Daiso Co. (Nishi-Ku, Osaka, Japan). Dimethylsulfoxide (DMSO), tetrahydrofuran (THF), TEMED, and TMPDA, purchased from Sigma-Aldrich Chemie (GmbH, Germany), were used as received. DEHEA, from Fluka Chemical Co. (Buchs, Switzerland), was used after distillation under reduced pressure. 2,2’-Azo-bis(isobutyronitrile) (AIBN), from Sigma-Aldrich, was purified by recrystallization three times from methanol. Potassium dichromate analytical grade and 1,5-diphenylcarbazide, purchased from Sigma-Aldrich Chemie (GmbH, Germany), were used as received. Vinylbenzyl chloride (VBC), purchased from Sigma-Aldrich, was used after distillation under reduced pressure (about 4 mm Hg), being kept at +4 °C. The synthesis of PVBC was performed by free radical polymerization of VBC with AIBN as initiator, at a concentration of 1.12 g/100 g VBC. The separation and characterization of PVBC have been presented in detail, elsewhere [47]. The PVBC used in this work had: M_w_ = 31,900 g/mol and M_w_/M_n_ = 1.74.

### 3.2. Synthesis of PSi/ANEX Composites

The synthesis of all PSi/ANEX composites was performed in three steps. In the first step, PVBC was introduced into the pores of PSi (1 or 2) by the sorption of polymer solution in THF, the concentration being 10 wt%. In the second step, PVBC was cross-linked with one of the diamine cross-linkers. Finally, the free -CH_2_Cl groups were reacted with DEHEA. The synthesis of the PSi1/ANEX1 is given as an example. 1 g of PVBC dissolved in 10 mL THF was added to 5 g PSi1 microspheres, and kept 2 h, at room temperature (RT). The polymer in excess was removed by a fast stirring (2 min) of silica microspheres in 8 mL THF. The supernatant was removed; the PVBC was precipitated in methanol p.a., separated by filtration, and kept in the air overnight. After that, the polymer was further dried 24 h in the oven at 40 °C, and weighed. The real amount of PVBC embedded into the silica pores was determined as 0.64 g. In the second step, silica containing PVBC was let overnight in air to remove THF, and then the crosslinking of PVBC with TEMED in a mole ratio of 0.2 moles TEMED:1 mole -CH_2_Cl was performed; in this case, the volume of the cross-linker was 0.2 mL in 9.8 mL THF added under fast stirring, and allowed to react at room temperature, 18 h, in a well closed bottle. Then, the THF was completely removed from the sample by evaporation (about 2 h). Finally, the -CH_2_Cl groups remained available after cross-linking were reacted with an excess of DEHEA (1.5 mL), in DMSO, at 60 °C, 15 h, the reaction being conducted in a horizontal plate thermostated shaker. To remove the solvent and the amine in excess, the composite microspheres were washed several times with methanol and water, as follows: first with methanol p.a. (1:1), under manual stirring, for 10 min; four times with distilled water (2:1), each 1 h; two times with methanol p.a., each 1 h. At this end, the composite was recovered and let overnight in the air to loose methanol. The PSi1/ANEX1 composite was then dried under vacuo, in the oven, four days at 40 °C. For the synthesis of PSi.2/ANEX2, the reaction steps were similar with those presented above, the only difference being that the amount of PVBC immobilized in 5 g of silica was 0.67 g, and the volume of the cross-linker (TMPDA) was 0.148 mL.

### 3.3. Equipments for Characterization

The organic material immobilized in porous silica was evaluated by TGA, under air at a heating rate of 10 °C/min with a device NETZSCH STA 449F1 (NETZSCH, Selb, Germany), with 5–8 mg composite. The structure of the PSi/ANEX composite sorbents was investigated by FTIR spectroscopy with a Bruker Vertex FTIR spectrometer (Bruker, Ettlingen, Germany), resolution of 2 cm^−1^, by KBr pellet technique, with 5 mg composite. The samples were scanned in the range of 4000–400 cm^−1^. The surface and interior morphology of the composite microspheres were investigated by SEM using an Environmental Scanning Electron Microscope (ESEM) (FEI Company, Hillsboro, OR, USA) type Quanta 200, coupled with EDX (SEM-EDX) for determination of the elemental composition. The specific surface area (S_sp_) of the composites along with pore volume were evaluated by N_2_ adsorption-desorption performed at 77 K by an Autosorb-1-MP surface area analyzer (NOVA, Quantachrome Company, Boynton beach, FL, USA). The isotherms of N_2_ adsorption/desorption were registered at the relative pressure p/p_o_ in the domain 0.01–1.0. The S_sp_ was evaluated from the linear portion of the adsorption isotherms by Brunauer-Emmet-Taylor (BET) method. The adsorbed volume was determined at values of the relative pressure p/p_o_ of about 0.95. Determination of the pH_PZC_ values, defined as the numeric value of pH where the potential is zero mV, of pristine silica and of the PSi/ANEX composites was performed by potentiometric titrations with a PCD 03 (Mütek, Germany) particle charge detector, between pH 2 and 12, using 0.01 mol·L^−1^ HCl and NaOH, respectively.

The evaluation of the water uptake (WU) of the PSi/ANEX composites was performed by immersing the composite samples in distilled water for 48 h. The WU (g/g) was calculated by Equation (6) [46]:

(6) $$\mathrm{WU}=\left(W_{t}-W_{d}\right)/W_{d}$$

where: W_d_ is the weight (g) of the dried microspheres, and W_t_ is the weight (g) of the hydrated composite at time t.

### 3.4. Sorption of Cr(VI) onto PSi/ANEX Composite in Batch Mode

Sorption experiments of Cr(VI) oxyanions from aqueous solution were performed in duplicates using a batch equilibrium procedure carried out on a water bath temperature controlled shaker (GFL 1083, Gemini BV, Apeldoorn, Nederland). The Cr(VI) aqueous solutions of various concentrations were prepared by suitable dilution of a stock solution of K_2_Cr_2_O_7_ with a concentration of 1000 mg Cr(VI)/L. The influence of the initial pH on the sorption Cr(VI) oxyanions at equilibrium, onto the PSi/ANEX composite sorbents was investigated in the range 2–7, using aqueous solutions with the initial concentration of 100 mg/L, adjusting the initial pH with 0.1 M HCl or 0.1 M NaOH. The samples collected at different contact times and at equilibrium were subsequently diluted, and the metal ion concentration was then evaluated by 1,5-diphenylcarbazide method, in acidic medium, using a UV-Vis spectrophotometer (Hitachi U-2001, λ = 540 nm, Triad Scientific Inc., Manasquan, NJ, USA) [25,48,58,59].

The amount of Cr(VI) ions sorbed by the PSi/ANEX samples, at equilibrium, q_e_ (mg Cr(VI)/g composite), was calculated with Equation (7):

(7) $$q_{e}=\frac{\left(C_{o}-C_{e}\right)V}{W}$$

where: C_o_ and C_e_ represent the metal ion concentration (mg/L) before and after the interaction with the composite sorbent, respectively; V—the volume of aqueous phase (L); W–the mass of composite sorbent (g).

Removal efficiency (RE, %), was evaluated using Equation (8):

(8) $$\mathrm{RE}=\frac{C_{o}-C_{e}}{C_{o}}\times 100$$

where: C_o_ and C_e_ have the same meaning as in Equation (7).

The sorption kinetics were investigated by adding 10 mL aqueous solution of K_2_Cr_2_O_7_ with a concentration of 100 mg/L in a flask containing about 0.01 g of dried PSi/ANEX composite, and allowed in contact at 23 °C, and 180 rpm for contact times increasing from 5 min to 8 h.

The experimental isotherms for the sorption of Cr(VI) oxyanions onto PSI/ANEX microspheres were determined at 23 °C, the metal concentration increasing from 10 to 1000 mg/L, at the initial pH of 4.0.

Desorption of the metal ions was performed with 0.1 M NaOH, at 23 °C, 6 h, under stirring.

Error analysis—two different error functions were used to determine the validity of the kinetic models and of the isotherm models fitted by the non-linear regression method: coefficient of determination (R^2^), and the non-linear Chi-square (χ^2^) test mathematically represented by Equation (9):

(9) $$\chi^{2}=\Sigma \frac{{\left(q_{e,\exp}-q_{e,\mathrm{cal}}\right)}^{2}}{q_{e,\mathrm{cal}}}$$

where q_e,exp_ and q_e,cal_ represent the experimental data (mg/g) and the data calculated by models (mg/g), respectively. If the data from a model are similar to the experimental data, χ^2^ is a small number, and if they strongly differ, χ^2^ is a big number.

## 4. Conclusions

The influence of the cross-linker type used in the synthesis of strong base anion exchangers (ANEX) embedded into the pores of two porous silica, a mesoporous one having S_sp_ of 95.097 m^2^/g (PSi1), and a macroporous having S_sp_ of 23.963 m^2^/g (PSi2), and of the silica morphology on the sorption capacity of Cr(VI) oxyanions in aqueous solution was deeply investigated in this work. The maximum sorption capacity was found when TEMED was used as cross-linker and PSi1 as support for ANEX, with a value of 311.2 mg Cr(VI)/g composite. Modeling of the sorption process showed that the sorption kinetics was well fitted with PSO kinetic model, and sorption at equilibrium was well described by Sips isotherm. The effect of pH on the sorption capacity and the thermodynamic parameters support ion exchange as the most probable mechanism of sorption. The novel composite sorbents are endowed with fast kinetics, the equilibrium of sorption established in about 60 min, and a remarkable reusability.

## Figures and Tables

**Table 1:** Kinetic parameters (m_sorbent_ = 10 mg, V_sol_ = 10 mL, C_in_ = 100 mg/L, temp. 23 °C, pH 4).

Kinetic Model	Kinetic Parameters	PSi.1/ANEX1	PSi.1/ANEX2	PSi.2/ANEX2
	q_e_, exp.	92.31	80.96	71.87
PFO	q_e, cal._ (mg/g)	88.9	78.08	69.97
	k_1_ (min^−1^)	0.0573	0.0553	0.0363
	R^2^	0.9531	0.955	0.976
	χ^2^	37.37	27.82	13.07
PSO	q_e calc._ (mg/g)	95.26	83.75	76.75
	k_2_ (g/mg·min)	9.494 × 10^−4^	0.001	6.74 × 10^−4^
	R^2^	0.9895	0.9895	0.9925
	χ^2^	8.4	0.46	4.1
Elovich	α (mg/g·min)	115.29	88.19	17.76
	β (g/mg)	0.0849	0.095	0.083
	R^2^	0.9758	0.9738	0.9631
	χ^2^	19.28	16.17	20.09
IPD model	k_id.1_, mg·g^−1^·min^−0.5^	5.274	4.888	5.63
	C_1_, mg·g^−1^	35.42	29.205	12.36
	R_1_^2^	0.9851	0.9928	0.9891

**Table 2:** Isotherm model parameters of Langmuir, Freundlich, Sips, Dubinin-Radushkevich (D-R), and Temkin models for the sorption of Cr(VI) onto the porous silica/ANEX composite sorbents, at 23 °C.

Isotherm	Isotherm Parameters	PSi.1/ANEX1	PSi.1/ANEX2	PSi.2/ANEX2
**Langmuir**	q_m_, mg/g	311.2	311.78	299.19
	K_L_, L/mg	0.01616	0.0115	0.00861
	R_L_	0.0062–0.712	0.0086–0.776	0.0115–0.2152
	R^2^	0.9979	0.9704	0.9532
	χ^2^	22	304	417
**Freundlich**	K_F_,mg^1−1/n^·L^1/n^·g^−1^	32.54	24.66	18.01
	1/n	0.3476	0.3816	0.4153
	R^2^	0.9269	0.8659	0.8496
	χ^2^	791	1378	1341
**Sips**	q_m,_ mg/g	301.55	265.19	242.19
	a_S_	0.01243	9.71 × 10^−4^	2.679 × 10^−4^
	1/n	1.086	1.7	1.937
	R^2^	0.9985	0.9979	0.9956
	χ^2^	16.4	21.4	38
**D-R**	q_DR._ mg/g	249.4	247.29	230.1
	β, mol^2^/kJ^2^	1.348 × 10^−4^	2.51 × 10^−4^	3.95 × 10^−4^
	E, kJ/mol	60.9	44.63	35.58
	R^2^	0.8910	0.9633	0.9747
	χ^2^	1171	377	226
**Temkin**	a_T_, L/mg	0.212	0.1057	0.0709
	b_T_, J/mol	40.52	35.69	35.15
	R^2^	0.9845	0.9604	0.9547
	χ^2^	168	407	403

**Table 3:** Comparison of the maximum sorption capacity (Langmuir model) of the porous silica/ANEX composite sorbents for Cr(VI) removal with other sorbents recently reported in literature.

Sorbent [ref]	Initial pH	T, °C	Sorbent Dose, g/L	q_m_, mg/g
Weakly basic anion exchange resin D301 modified with poly-epichlorohydrin-dimethylamine (EPIDMA) [22]	2	25	0.6	194
Macroporous copolymers of poly(glycidyl methacrylate-co-ethyleneglycol dimethacrylate functionalized with hexamethylene diamine (GMA-60 HD) [23]	2	25	10	156
Composites with strong base anion exchangers embedded into chitosan/poly(vinyl amine) cryogels [24]	5.5	25	1.25	295.92
Macroporous styrene-divinylbenzene matrix, functional group—^+^N(CH_2_CH_3_)_2_CH_2_CH_2_OH [25]	5.5	25	1	313.81
poly(N1,N1,N3,N3-tetraallylpropane-1,3-diaminium chloride) [26]	3–7	20	1	273.17
Macroporous anion exchange resin D301 [27]	3	27	1.7	151.52
Gels of poly[2-(dimethylamino)ethyl methacrylate] with bromethan [28]	4.5	25	1	210.5
Chitosan modified with cinnamaldehyde nanocomposites [32]	2	25	3	61.35
Hydroxyapatite microfibrillated cellulose composite [35]	5	25	0.8	114.8
Polyvinylpyrrolidone intercalated molybdenum disulfide [36]	5	25	0.5	142.2
Mesoporous silica MCM-41/PMMA [41]	2	25		20
Polypyrrole-polyaniline nanofibers [49]	2	25	1	227
Polyacrylonitrile/polypyrole core/shell nanofiber mat [55]	2	25	3.33	61.8
Porous silica PSi.1/ANEX1 composite [this study]	4	23	1	311.8

**Table 4:** Thermodynamic parameters for the sorption of Cr(VI) oxyanions onto the porous silica/ANEX composite sorbents.

Sorbent	ΔH, kJ/mol	ΔS, kJ/mol.K	ΔG, kJ/mol				
			298 K	303 K	308 K	313 K	318 K
PSi.1/ANEX1	33.4	0.214	−30.37	−31.44	−32.51	−33.58	−34.65
PSi.1/ANEX2	33.35	0.209	−28.93	−29.98	−31.02	−32.07	−33.11
PSi.2/ANEX2	32.96	0.205	−28.13	−29.16	−29.79	−31.21	−32.23

## Supplementary Materials

The following are available online at https://www.mdpi.com/1420-3049/25/5/1249/s1. Figure S1: N_2_ adsorption–desorption isotherms on the pristine silica (a and b) and on the PSi/ANEX composites (c and d); distribution of pore diameter (BJH) for the pristine silica (e and f) and for PSi1/ANEX2 (g) and PSi/2/ANEX2 (h), Figure S2: FTIR spectra of pristine PSi1 and PSi2, Figure S3: Comparative EDX profiles of the PSi/ANEX composites, Figure S4: TG-DTG curves of the pristine PSi2. Figure S5: Calibration curve for spectrophotometric determination of Cr(VI) in aqueous solutions. Table S1: Water uptake (WU) of the PSi/ANEX composites compared with pristine porous silica, Table S2: Non-linear forms of the kinetic models fitted on the kinetic data, Table S3, Non-linear forms of the isotherm models applied in this work.
