# Erratum: Dragan, E.S., et al., Contribution of Cross-Linker and Silica Morphology on Cr(VI) Sorption Performances of Organic Anion Exchangers Embedded into Silica Pores. *Molecules* 2020, *25*, 1249

**DOI:** 10.3390/molecules25122736

**Published:** 2020-06-12

**Authors:** Ecaterina Stela Dragan, Doina Humelnicu

**Affiliations:** 1 “Petru Poni” Institute of Macromolecular Chemistry, Grigore Ghica Voda Alley 41 A, 700487 Iasi, Romania; 2 Faculty of Chemistry, “Al. I. Cuza” University of Iasi, Bd. 11 Carol I, 700506 Iasi, Romania; doinah@uaic.ro

The authors wish to make a change to the published paper [1]. In the original manuscript, there is a mistake in the words “Brunauer-Emmet-Taylor (BET)” on line 13 in Section 3.3 “Equipments for Characterization”. The correct words are “Brunauer–Emmet–Teller (BET)”.

The authors apologize for any inconvenience caused, and the change does not affect the scientific results. The manuscript will be updated, and the original will remain online on the article webpage at https://www.mdpi.com/1420-3049/25/5/1249.
