# Supplementary material for: Contribution of Cross-Linker and Silica Morphology on Cr(VI) Sorption Performances of Organic Anion Exchangers Embedded into Silica Pores
Source: Molecules. 2020 Mar 10;25(5):1249. doi: 10.3390/molecules25051249 (PMC7179461; doi:10.3390/molecules25051249)
Supplement: Supplementary file 1 [file molecules-25-01249-s001.pdf]

Supplementary Materials

Contribution of Cross-Linker and Silica Morphology on Cr(VI) Sorption Performances of Organic Anion Exchangers Embedded into Silica Pores

Ecaterina Stela Dragan [1,*] and Doina Humelnicu [2]

[1] "Petru Poni" Institute of Macromolecular Chemistry, Grigore Ghica Voda Alley 41 A, 700487 Iasi, Romania
[2] Faculty of Chemistry, "Al. I. Cuza" University of Iasi, Bd. 11 Carol I, 700506 Iasi, Romania; doinah@uaic.ro
* Correspondence: sdragan@icmpp.ro; Tel.: +40-232-217454

Figure S1. N_2 adsorption–desorption isotherms on the pristine silica (a and b) and on the PSi/ANEX composites (c and d); distribution of pore diameter (BJH) for the pristine silica (e and f) and for Psi1/ANEX2 (g).

Figure S2. FTIR spectra of pristine PSi1 and PSi2.

Figure S3. Comparative EDX profiles of the PSi/ANEX composites.

Figure S4. TG-DTG curves of the pristine PSi2.

Figure S5. Calibration curve for spectrophotometric determination of Cr(VI) in aqueous solutions.

Table S1. Water uptake (WU) of the PSi/ANEX composites compared with pristine porous silica.

Table S2. Non-linear forms of the kinetic models fitted on the kinetic data.

Table S3. Non-linear forms of the isotherm models applied in this work [6–8].

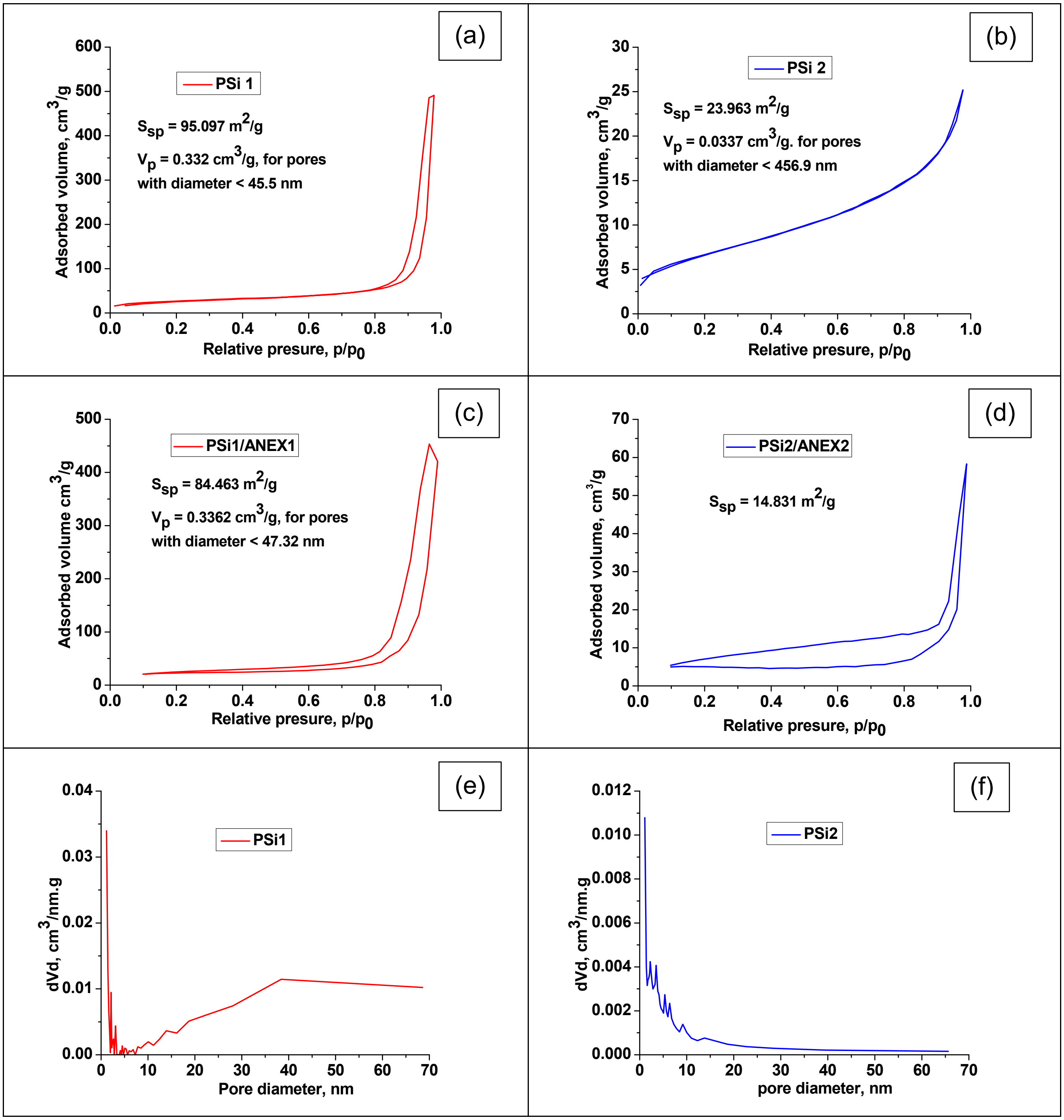
(a)
PSi 1
Ssp = 95.097 m2/g
Vp = 0.332 cm3/g, for pores
with diameter < 45.5 nm
Adsorbed volume, cm3/g
Relative presure, p/p0
(b)
PSi 2
Ssp = 23.963 m2/g
Vp = 0.0337 cm3/g. for pores
with diameter < 456.9 nm
Adsorbed volume, cm3/g
Relative presure, p/p0
(c)
PSi1/ANEX1
Ssp = 84.463 m2/g
Vp = 0.3362 cm3/g, for pores
with diameter < 47.32 nm
Adsorbed volume cm3/g
Relative presure, p/p0
(d)
PSi2/ANEX2
Ssp = 14.831 m2/g
Adsorbed volume, cm3/g
Relative presure, p/p0
(e)
PSi1
dVd, cm3/nm.g
Pore diameter, nm
(f)
PSi2
dVd, cm3/nm.g
pore diameter, nm

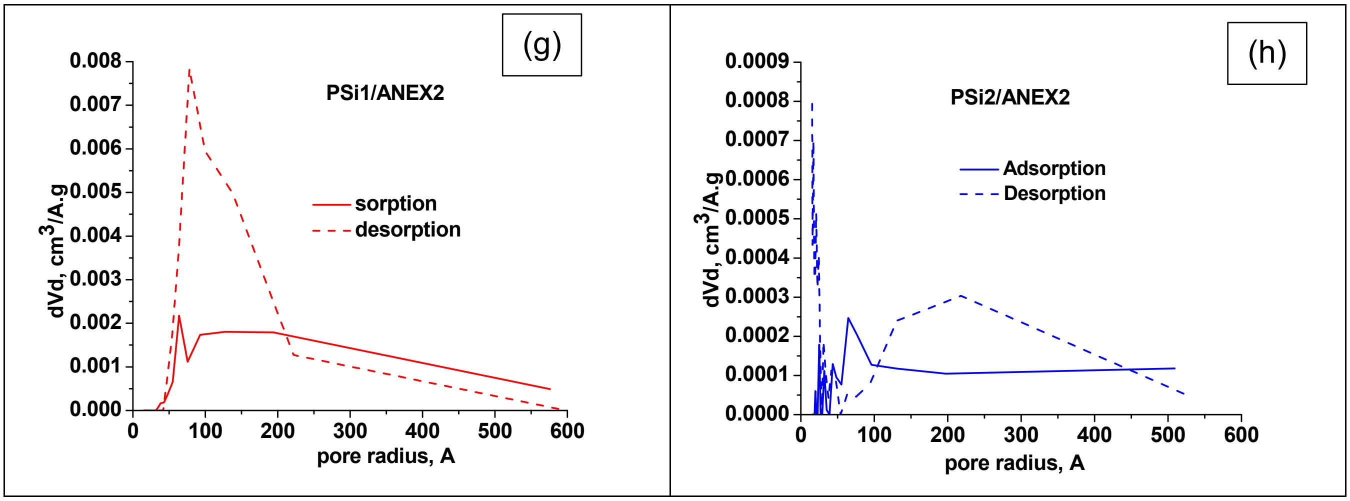

Figure S1. N_2 adsorption–desorption isotherms on the pristine silica (a and b) and on the PSi/ANEX composites (c and d); distribution of pore diameter (BJH) for the pristine silica (e and f) and for PSi1/ANEX2 (g) and PSi/2/ANEX2 (h).

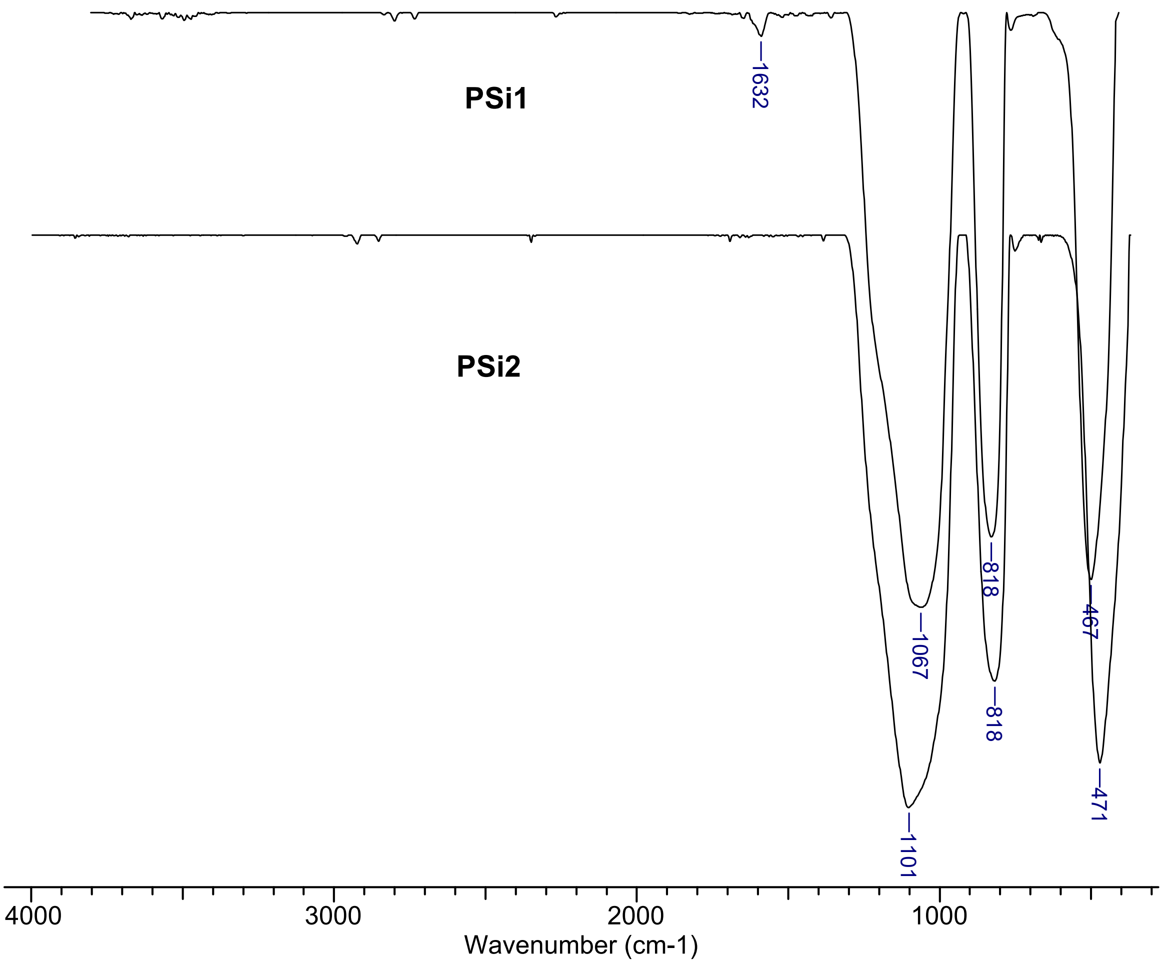

Figure S2. FTIR spectra of pristine PSi1 and PSi2.

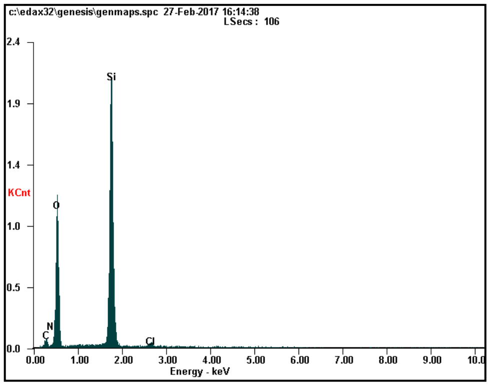

PSi1/ANEX1

Element	Wt%	At%
CK	14.09	20.43
NK	03.85	04.79
OK	51.24	55.77
SiK	30.05	18.63
ClK	00.77	00.38
Matrix	Correction	ZAF

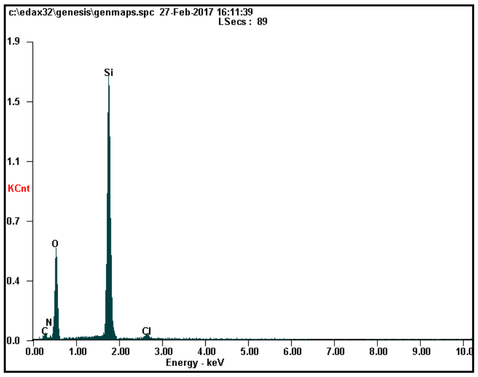

PSi1/ANEX2

Element	Wt%	At%
CK	15.20	22.66
NK	02.95	03.77
OK	44.76	50.09
SiK	35.78	22.81
ClK	01.32	00.66
Matrix	Correction	ZAF

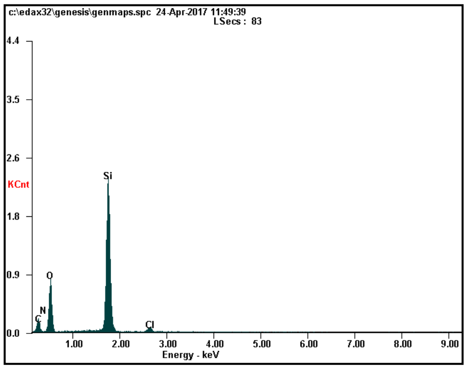

PSi2/ANEX2

Element	Wt%	At%
CK	29.24	39.84
NK	03.32	03.87
OK	38.91	39.80
SiK	27.27	15.89
ClK	01.27	00.58
Matrix	Correction	ZAF

Figure S3. Comparative EDX profiles of the PSi/ANEX composites.

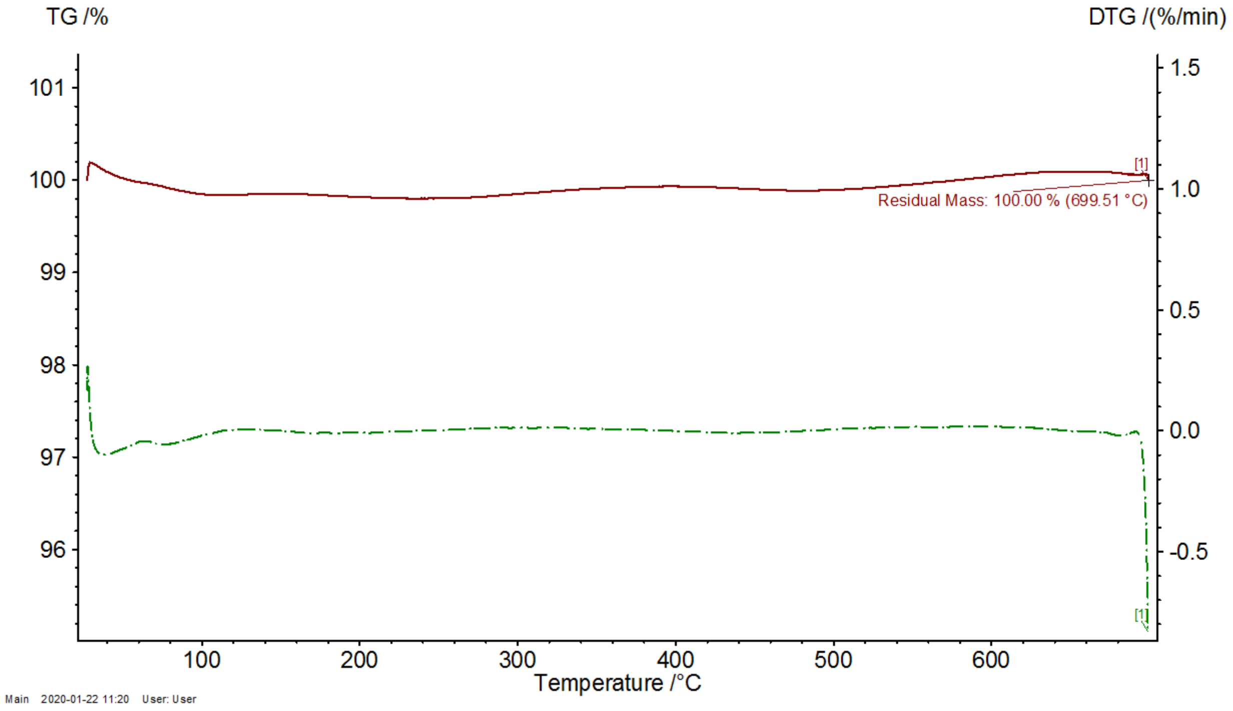

Figure S4. TG-DTG curves of pristine PSi2.

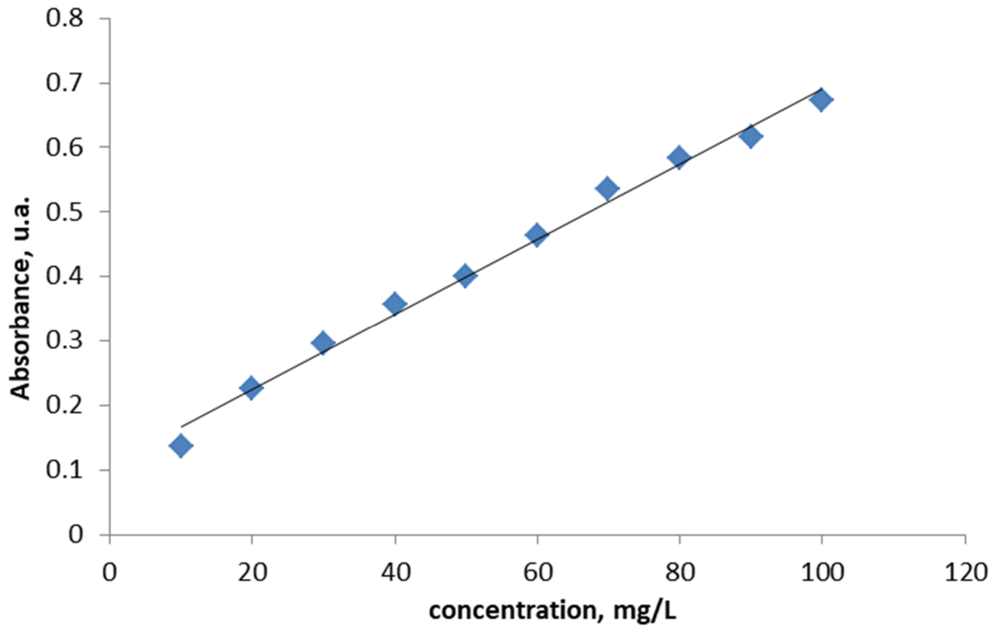

Figure S5. Calibration curve for spectrophotometric determination of Cr(VI) in aqueous solutions.

Using the linear regression method, the values of LOD 6.708 mg/L, LOQ 20.327 mg/L, calibration sensitivity of 0.0058, and correlation coefficient 0.9915 were obtained.

Table S1. Water uptake (WU) of the PSi/ANEX composites compared with pristine porous silica.

Sample	PSi1	PSi2	PSi1/ANEX1	PSi1/ANEX2	PSi2/ANEX2
WU, g/g	1.718	1.541	0.9271	1.281	1.176

Table S2. Non-linear forms of the kinetic models fitted on the kinetic data.

Kinetic model	Equation	Parameters	References
Pseudo-first-order	$q_t = q_e\ (1- e^{-k1t})$	q_e and q_t - the amount of Cr(VI) ions sorbed at equilibrium (mg/g) and at time t, respectively k_1 - the rate constant of the PFO kinetic model (min^{-1}).	[1]
Pseudo-second-order	$q_t = \frac{k_2 q_e{}^2 t}{1 + k_2 q_e t}$	q_e and q_t have the same meaning as in the PFO model k_2 - the rate constant of PSO kinetic model (g mg^{-1} min^{-1}).	[2]
Elovich	$q_t = \frac{1}{\beta} \ln (1 + \alpha \cdot \beta \cdot t)$	α (mg/g min–1) is the initial sorption rate and β (g mg–1) is correlated to the degree of surface coverage and activation energy for chemisorption	[3,4]
Intra-particle diffusion (IPD)	$q_t = k_{id} \cdot t^{0.5} + C_i$	k_{id} - the intra-particle diffusion rate constant (g mg^{-1} $min^{-0.5}$) C_i - constant that gives an idea about the effect of boundary layer thickness (mg/g)	[4]

The Lagergren rate equation considers the adsorption rate proportional with the difference between the equilibrium adsorption capacity and the adsorbed amount. The Ho and Mc. Kay pseudo-second-order

(PSO) kinetic model is based on the assumption that the rate-limiting step is chemisorption, involving valence forces through sharing or exchange electrons between adsorbent and sorbate. The Elovich model assumes that activated sorption is dominant on heterogeneous surfaces and that the adsorption rate decreases in time. Intra-particle diffusion (IPD) assumes that internal diffusion of adsorbate into the surface of the adsorbent is related only to the mass transfer resistance and determines the adsorption rate of the liquid system, a linear regression of plots which passes through the origin (C = 0) supports the IPD as the sole rate-determining step.

Table S3. Non-linear forms of the isotherm models applied in this work [6-8].

Isotherm/Ref	Equation	Isotherm parameters
**Langmuir**	$q_e = \frac{q_m K_L C_e}{1 + K_L C_e}$	q_m - the theoretical limit of adsorption when the monolayer surface is fully covered with metal ions (mg/g); K_L - the Langmuir constant (L/mg);
**Freundlich**	$q_e = K_F C_e^{1/n}$	K_F - Freundlich constant ($mg^{1-1/n}.L^{1/n}.g^{-1}$); n - indicates the distribution of active sites related to the surface heterogeneity; the larger the n value, more heterogeneous is the system
**Sips**	$q_e = \frac{q_m a_S C_e^{1/n}}{1 + a_S C_e^{1/n}}$	q_m - the monolayer adsorption capacity (mg/g); a_S - the Sips constant related to the energy of adsorption; $1/n$ – values close to zero indicate heterogeneous sorbent, while values closer to 1 indicate a homogeneous distribution of binding sites; $1/n$ = 1, Sips isotherm coincides with Langmuir equation
**D-R**	$q_e = q_{DR} \exp\{-\beta[RT \ln(1 + \frac{1}{C_e})]^2\}$	q_{DR} - the maximum adsorption capacity of the metal ion (mg/g); β - the D-R isotherm constant (mol^2/kJ^2)
**Temkin**	$q_e = \frac{RT}{b_T} \ln a_T C_e$	b_T - the Temkin constant related to the heat of sorption (kJ/mol); a_T - the equilibrium binding constant corresponding to the maximum binding energy (L/mg); R is the gas constant (8.314 x 10^{-3} kJ mol^{-1} K^{-1}); T is the absolute temperature (K)

Langmuir isotherm is applicable to homogeneous adsorption and is based on the following assumptions: (1) all adsorption sites are identical; (2) each site retains one molecule of the given compound; (3) all sites are energetically and sterically independent of the adsorbed quantity. The feasibility of adsorption in a certain concentration range can be expressed in terms of a dimensionless constant R_L, called constant separation factor or equilibrium parameter:

$$R_L = \frac{1}{1 + K_L C_i}$$

where: K_L is the Langmuir adsorption constant (L/mg), and C_i is the initial concentration of sorbate (mg/L).

The adsorption is: unfavorable when $R_L > 1$; linear when $R_L = 1$; favorable when $0 < R_L < 1$; irreversible when $R_L = 0$.

Freundlich isotherm is an empirical equation used to model the adsorption process on heterogeneous surfaces, suggesting that binding sites are not equivalent. Sips isotherm is a combination of the Langmuir and Freundlich isotherm models. At low sorbate concentrations, Sips isotherm is equivalent with the Freundlich isotherm, while at high sorbate concentrations, it predicts a monolayer adsorption capacity characteristic to the Langmuir isotherm. The D-R isotherm was applied to distinguish between physical and chemical sorption. The Temkin isotherm equation assumes that the heat of adsorption of the molecules decreases linearly with coverage due to sorbent-sorbate interactions, and that the adsorption is characterized by a uniform distribution of the bonding energies.

References:

1. Lagergreen, S. Kungliga Svenska Vetenskapsakademiens. Handlingar. **1898**, *24*, 1-39.
2. Ho, Y.S.; McKay, G. The kinetics of sorption of basic dyes from aqueous solution by sphagnum moss peat. *Can. J. Chem. Eng*. **1998**, *76*, 822-827.
3. Juang, R.S.; Chen, M.L. Application of the Elovich equation to the kinetics of metal sorption with solvent-impregnated resins. *Ind. Eng. Chem. Res*. **1997**, *36*, 813-820.
4. Ho Y.S.; McKay, G. Application of Kinetic Models to the Sorption of Copper(II) on to Peat. *Ads. Sci. Technol.* **2002**, 20, 797-815.
5. Weber, J.W.J.; Morris, J.C. Kinetics of adsorption on carbon from solution. *J. San. Eng. Div. Proceed. Am. Soc. Civ. Eng*. **1963**, *89*, 31-60.
6. Foo, K.Y.; Hameed, B.H. Insights into the modeling of adsorption isotherm systems. *Chem. Eng. J.* **2010**, *156*, 2-10.
7. Srivastava, Y.C.; Swamy, M.M.; Mall, I.D.; Prasad, B.; Mishra, I.M. Adsorptive removal of phenol by bagasse fly ash and activated carbon: Equilibrium, kinetics and thermodynamics. *Colloids Surf. A* **2006**, *272*, 89-104.
8. Apopei, D.F.; Dinu, M.V.; Trochimczuk, A.; Dragan, E.S. Sorption isotherms of heavy metal ions onto semi-interpenetrating polymer network cryogels based on polyacrylamide and anionically modified potato starch. *Ind. Eng. Chem. Res.* **2012**, *51*, 10462-10471.
